# Identification and characterization of a novel human adenovirus type HAdV-D116

**DOI:** 10.3389/fmicb.2025.1566316

**Published:** 2025-05-07

**Authors:** Menglan Zhou, Wenjing Chen, Dong Zhang, Shicheng Ma, Mange Liu, Lili Ren, Jiayu Guo, Yi Gao, Minya Lu, Huiting Su, Ying Zhao, Yingchun Xu, Qiwen Yang

**Affiliations:** ^1^Department of Laboratory Medicine, State Key Laboratory of Complex Severe and Rare Diseases, Peking Union Medical College Hospital, Chinese Academy of Medical Sciences and Peking Union Medical College, Beijing, China; ^2^Beijing Key Laboratory for Mechanisms Research and Precision Diagnosis of Invasive Fungal Diseases, Beijing, China; ^3^Vision Medicals Center for Infectious Diseases, Guangzhou, China; ^4^Department of Emergency, Peking Union Medical College Hospital, Chinese Academy of Medical Sciences and Peking Union Medical College, Beijing, China; ^5^Department of Neurology, Peking Union Medical College Hospital, Chinese Academy of Medical Sciences and Peking Union Medical College, Beijing, China; ^6^Key Laboratory of Respiratory Disease Pathogenomics, Chinese Academy of Medical Sciences and Peking Union Medical College, Beijing, China; ^7^Key Laboratory of Pathogen Infection Prevention and Control, Peking Union Medical College, Ministry of Education, Beijing, China

**Keywords:** encephalitis, human adenovirus, X-linked agammaglobulinemia, metagenomic next-generation sequencing, HAdV-D116, AlphaFold2

## Abstract

**Introduction:**

Human adenovirus infections are typically associated with acute respiratory infection, keratoconjunctivitis, acute cystitis, hepatitis, and gastroenteritis, while central nervous system (CNS) related infections are rarely reported.

**Methods:**

In this study, a novel human adenovirus was identified in the cerebrospinal fluid from an encephalitis patient with X-linked agammaglobulinemia via metagenomic next-generation sequencing (mNGS). Probe capture enrichment sequencing and PCR validation further confirmed the presence of this adenovirus in the patient’s cerebrospinal fluid.

**Results:**

Whole-genome analysis classified the virus within the Human mastadenovirus D species, revealing an approximately 2000 bp deletion in the E3 gene that resulted in the loss of CR1-gamma and RID-alpha regions and the formation of a novel open reading frame (ORF). The penton base, hexon, and fiber genes were identified as P33H28F71, designating this virus as a novel type, subsequently named HAdV-D116 by the Human Adenovirus Working Group. Recombination analysis suggested that HAdV-D116 is a recombinant strain derived from HAdV-D33, HAdV-D28, and HAdV-D71. Structural analysis of the fiber-knob domain indicated that HAdV-D116 likely uses sialic acid as a receptor.

**Discussion:**

The unique genomic features of HAdV-D116, combined with the patient’s immunodeficiency, are proposed to contribute to its possible CNS infectivity. The discovery of HAdV-D116 expands our understanding of human adenovirus tropism and underscores the need for vigilance regarding the emergence of novel adenovirus-related CNS infections.

## Introduction

Human adenoviruses (HAdVs) are non-enveloped, double-stranded DNA viruses that cause a wide range of infections, including acute respiratory illness, keratoconjunctivitis, acute cystitis, hepatitis, and gastroenteritis (Zhao et al., 2022). To date, over 100 genotypes have been identified, classified into seven species (A-G) (Mennechet et al., 2019). HAdVs associated with central nervous system (CNS) diseases are rarely reported and typically occur in infants or immunocompromised patients, most frequently involving species B or C (Zhao et al., 2022; Tunkel et al., 2019; Okamoto et al., 2004; Chatterjee et al., 2000). Although adenovirus infections of the CNS are rare, they pose significant clinical challenges, particularly in immunocompromised patients, where they can result in substantial mortality rates and cause long-term neurological sequelae in survivors (Echavarria, 2008). Persistent HAdV infections in immunocompromised individuals, such as those with acquired immune deficiency syndrome (AIDS), can result in severe clinical outcomes (Lion, 2014). Currently, therapeutic options for adenovirus infections in immunocompromised individuals remain severely limited, with no regulatory approved antiviral agents specifically targeting adenoviruses. Management typically relies on supportive care measures, while the absence of standardized treatment protocols and limited understanding of adenovirus infection mechanisms in the central nervous system further complicates clinical decision-making (Matthes-Martin et al., 2013).

HAdVs contribute to a variety of disease conditions, including respiratory, gastrointestinal, genitourinary, and ocular infections. Compared to HAdV-A, B, C which were commonly associated with acute respiratory infections, HAdV-D caused gastrointestinal tract infections and epidemic keratoconjunctivitis more often (Ismail et al., 2019; Mao et al., 2022; Mundy et al., 2023). HAdV-D is the most genetically diverse and exhibits a higher tendency for recombination compared to other species (Fujimoto et al., 2014). Certain types, including HAdV-D8, HAdV-D37, HAdV-D53, HAdV-D54, HAdV-D56, and HAdV-D64, have been linked to epidemic keratoconjunctivitis (Ismail et al., 2019; Rajaiya et al., 2021). Moreover, newly emerging members of HAdV-D have been identified in patients with AIDS and other immunocompromised conditions, suggesting that impaired host immune responses may facilitate the recombination and evolution of novel HAdV strains (Alissa Alkhalaf et al., 2014; Kajon et al., 2014; Hage et al., 2017).

HAdVs were initially classified by serological methods, with types 1 to 51 identified through this approach (Ishiko et al., 2008; Jones et al., 2007; Ghebremedhin, 2014). In 2011, the Human Adenovirus Working Group^1^ recommended using whole-genome sequencing for the characterization and naming of human adenoviruses (Seto et al., 2011). Prior to this recommendation, HAdV-G52, HAdV-D53, HAdV-D54, HAdV-B55, and HAdV-D56 had already been classified using whole-genome sequencing and bioinformatic analysis, with HAdV-G52 being the first type identified through these methods (Jones et al., 2007; Seto et al., 2011; Robinson et al., 2011a; Walsh et al., 2010; Walsh et al., 2009). Since then, all newly identified types from HAdV-52 to the current HAdV-116 have been characterized by DNA sequencing and bioinformatics. Recombination is a recognized feature of HAdV evolution, and new recombinant types are classified based on significant genomic, biological, or pathogenic differences from existing types, as determined by analysis of the penton base, hexon, and fiber genes (Seto et al., 2011).

The HAdV capsid is composed of three major proteins: hexons, penton bases, and fibers. Several attachment receptors for HAdVs have been identified, including the Coxsackie and Adenovirus Receptor (CAR), CD46, desmoglein-2 (DSG-2), and the glycans GD1a and polysialic acid (Stasiak and Stehle, 2020; Zhang and Bergelson, 2005). Typically, HAdV entry into host cells involves the fiber knob anchoring the virus to primary cellular receptors, while the penton base engages secondary co-receptors to facilitate viral entry (Stasiak and Stehle, 2020; Zhang and Bergelson, 2005). Recently, a direct interaction between the hexon protein and CD46 has also been reported as an alternative entry mechanism, particularly for species D adenoviruses (Persson et al., 2021). The diversity of HAdV receptors contributes to the broad tropism of these viruses (Stasiak and Stehle, 2020). For example, HAdV-D26, a known causative agent of epidemic keratoconjunctivitis (EKC), utilizes sialic acid-bearing glycans as a primary entry receptor via the fiber knob (Metsky et al., 2019). In contrast, HAdV-D56, which is associated with neonatal respiratory fatality and adult EKC, enters host cells through a direct interaction between the hexon protein and CD46 (Persson et al., 2021). Moreover, at least 16 different HAdV-D types, including HAdV-D26 and HAdV-D28, have been shown to interact directly with CD46 through the hexon protein (Persson et al., 2021). Despite these findings, the mechanisms by which HAdV-D binds to receptors and mediates cell entry during CNS infections remain unexplored.

In this study, we report the identification of a novel human adenovirus, HAdV-D116, detected in the cerebrospinal fluid of a patient with encephalitis and X-linked agammaglobulinemia (XLA) using metagenomic next-generation sequencing (mNGS). A combined genomic and bioinformatic approach was employed to elucidate the evolutionary characteristics of this novel adenovirus.

## Materials and methods

### Metagenomic next-generation sequencing

Cerebrospinal fluid and blood samples from the patient were collected for mNGS. The study was approved by the Institutional Review Board of Peking Union Medical College Hospital. Written informed consent was obtained from the patient. CSF was collected via standard lumbar puncture under aseptic conditions. Approximately 5 mL of CSF was collected in sterile tubes and immediately transported to the laboratory on ice. The sample was centrifuged at 1,500 × *g* for 10 min at 4°C to remove cellular debris. The supernatant was aliquoted and stored at −80°C until further processing. For blood samples, 5 mL of peripheral blood was collected in EDTA tubes and processed within 2 h of collection. Plasma was separated by centrifugation at 1,600 × *g* for 10 min to remove cells and debris. The processed plasma was stored at −80°C until DNA/RNA extraction. Microbial DNA was extracted using the QIAamp^®^ UCP Pathogen DNA Kit (Qiagen, Valencia, CA, USA) according to the manufacturer’s protocol. Total RNA from these samples was extracted using QIAamp® Viral RNA Kit (Qiagen, Valencia, CA, USA), then subjected to human rRNA depletion by application of Ribo-Zero rRNA Removal Kit (Illumina, San Diego, CA, USA), following the manufacturer’s instructions. After removing the contaminating DNA via DNase I, cDNA was generated using reverse transcriptase and dNTPs. Subsequently, both DNA and cDNA preparations were combined, and libraries were constructed using the Nextera XT DNA Library Prep Kit (Illumina) and their quality was evaluated with the AGILENT Qubit dsDNA HS Assay kit on an Agilent 2100 bioanalyzer. Once library quality was confirmed, sequencing was performed on the Illumina NextSeq 550 platform using a single-end 75 bp sequencing strategy. For targeted metagenomic sequencing, an additional hybrid capture step was employed post-library construction. Specifically, 750 ng of each library was subjected to hybrid capture-based enrichment of microbial probes (SeqCap EZ Library, Roche, USA), with probes designed via the CATCH pipeline (Metsky et al., 2019).

### Quality control and microbial identification

The obtained sequencing data were subjected to quality control using fastp (v0.20.0) (Chen, 2023), which included adapter trimming, removal of low-quality reads, low-complexity reads, and sequences shorter than 40 bp. Subsequently, human reads were removed by aligning the reads to the human reference genome (hg38) using BWA (v0.7.17-r1188) (Li, 2013). The remaining reads were then aligned to a microbial database curated from the NCBI RefSeq, which includes representative genomes of bacteria, viruses, fungi, and protozoa. Taxonomic classification of species was determined by reads specifically mapped to the reference genomes with over 96% (92% for virus) in nucleotide identity. The aligned reads were further validated through a BLASTn search against the NCBI nucleotide (nt) database. Additionally, after the removal of human reads, the remaining reads were assembled using SPAdes (v3.13.0) (Prjibelski et al., 2020), and the resulting contigs were identified by conducting a BLASTn search against the nt database.^2^

### Phylogenetic tree analysis and genome annotation

An assembled sequence, identified as human adenovirus through BLASTn, along with 112 known HAdV genomes obtained from NCBI, were used to construct a phylogenetic tree. Multiple alignments were built using Clustal-Omega (v1.2.4) (Sievers and Higgins, 2018). Phylogenetic trees were subsequently constructed with RAxML-NG (v1.2.0) (Kozlov et al., 2019), applying the maximum likelihood method with 1,000 bootstrap replicates and the GTR + I + G4 model, as identified by ModelTest-NG (v0.1.7) (Darriba et al., 2020). Visualization of the phylogenetic tree was performed using GraPhlAn (v1.1.3) (Asnicar et al., 2015). Open reading frames (ORFs) of the assembled sequence were predicted and annotated using ORFfinder (v0.4.3)^3^ with the genetic code set to standard and ATG as the only start codon, and VIGOR (v4.1.20200702) (Wang et al., 2010), which utilized the coding sequences (CDSs) of HAdV-D60 as a reference, as it shared the closest clade with the assembled sequence in the phylogenetic tree. ORF annotations were validated by performing a BLASTp search against the nucleotide database. The genome organization was illustrated using Python scripts and manually curated diagrams based on the annotation results. Amino acid sequences of penton base, hexon, and fiber-knob were extracted from the genomes, aligned using MUSCLE (v5.2) (Edgar, 2022), and phylogenetic trees were constructed using IQ-TREE (v2.2.2.6) (Minh et al., 2020) with 1,000 ultrafast bootstraps, automatic model detection, and default parameters.

### Genome similarity and BootScan analysis

HAdV-D116 was compared with HAdV-D33, HAdV-D28, and HAdV-D71 using SimPlot (v3.5.1) (Lole et al., 1999). For similarity analysis, HAdV-D116 was used as the query, with a window size of 500 bp and a step size of 20 bp, while all other parameters were set to default. BootScan analysis was also conducted using HAdV-D116 as the query under the same window and step size settings.

### Deletion analysis

Non-human reads were aligned to the genomes of HAdV-D60 and HAdV-D116 to analyze and confirm genome coverage and the presence of deletions. These deletions were subsequently validated through PCR and Sanger sequencing using the following primer sequences: forward 5′-GCCAGTTACATGGCTTGGTG-3′ and reverse 5′-GCAGGAGCAGACCATGACTA-3′, targeting a 205 bp sequence (genome positions 27,814–28,018).

### Structural analysis

Protein structure predictions were generated using ColabFold (v1.5.3).^4^ Protein structure alignment was performed using the RCSB service,^5^ and structure visualizations were generated using PyMOL (v2.5.5).

### Genomic polymorphism analysis

Nucleotide diversity across the entire genome was calculated using DnaSP (v6.12.03) (Rozas et al., 2017) based on whole-genome alignments for each HAdV species. The average number of nucleotide differences per site was plotted using a 200 bp sliding window with a step size of 20 bp.

## Results

### Clinical presentation

**Figure 1:**
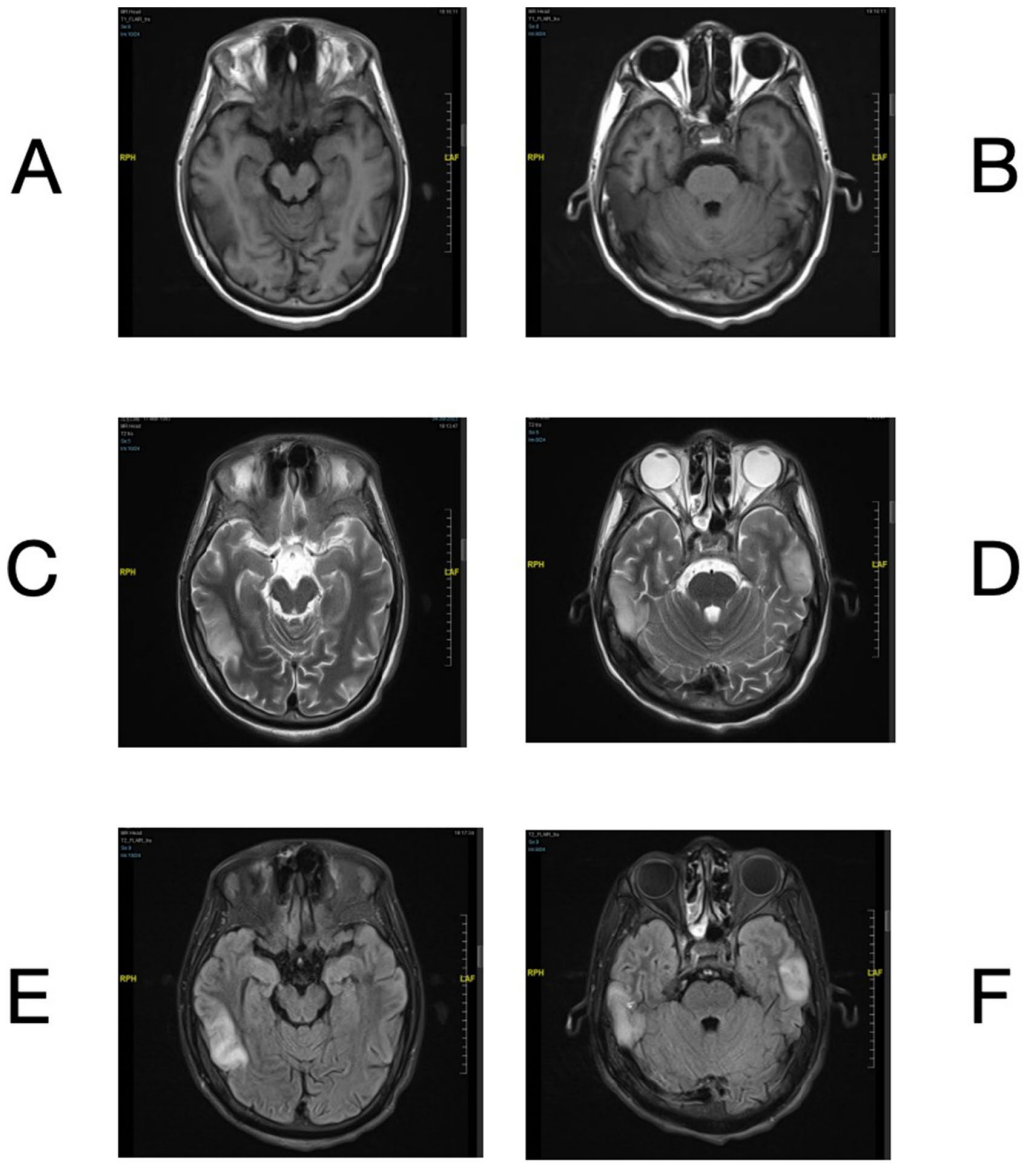
Brain MRI of bilateral temporal lobe lesions. Brain MRI showed T1 hypointensity **(A,B)** and T2 hyperintensity **(C,D)** in bilateral temporal lobes. The lesions exhibited hyperintensity on the FLAIR sequence **(E,F)**.

A 30-year-old man presented to the emergency room on August 1st, 2023, with a sudden onset of dyslexia, logasthenia, and an expressive language disorder that began 3 days prior to admission. He did not report any headache, dizziness, hallucinations, limb numbness, or psychiatric disorders. Cranial magnetic resonance imaging (MRI) revealed multiple abnormal signals in the bitemporal lobes (Figures 1A–F). His vital signs were generally normal, except for a fever of 38°C. Physical examination showed negative meningeal irritation signs but indicated severe vision impairment, with a visual acuity of 0.1 in the left eye and only light perception in the right eye. A lumbar puncture performed the next day revealed an increased white blood cell count with a predominance of monocytes, as well as slightly elevated protein levels in the cerebrospinal fluid (CSF). Significantly elevated levels of interleukin (IL)-6, IL-8, and IL-10 were also noted (Supplementary Table S1). Tests for traditional bacteria and fungi culture and autoimmune encephalitis-related antibodies in the CSF were negative. Additional tests screening for cytomegalovirus (CMV) DNA, Epstein–Barr virus (EBV) DNA, polyomaviruses BK and JC DNA in the CSF, antibodies of CMV, EBV, rubella virus, parvovirus B19 and herpes simplex virus in the blood all yielded negative results. To elucidate the etiology of the patient’s condition, CSF and blood samples were collected and subjected to mNGS for pathogen screening. Whole exome sequencing previously revealed a novel but deleterious mutation, c.1880A > G (p.Tyr627Cys), in exon 17 of the BTK gene on the X chromosome, which was confirmed to have been inherited from his mother, a heterozygote. This finding supported his diagnosis of X-linked agammaglobulinemia (XLA).

### Metagenomic next-generation sequencing analysis

**Figure 2:**
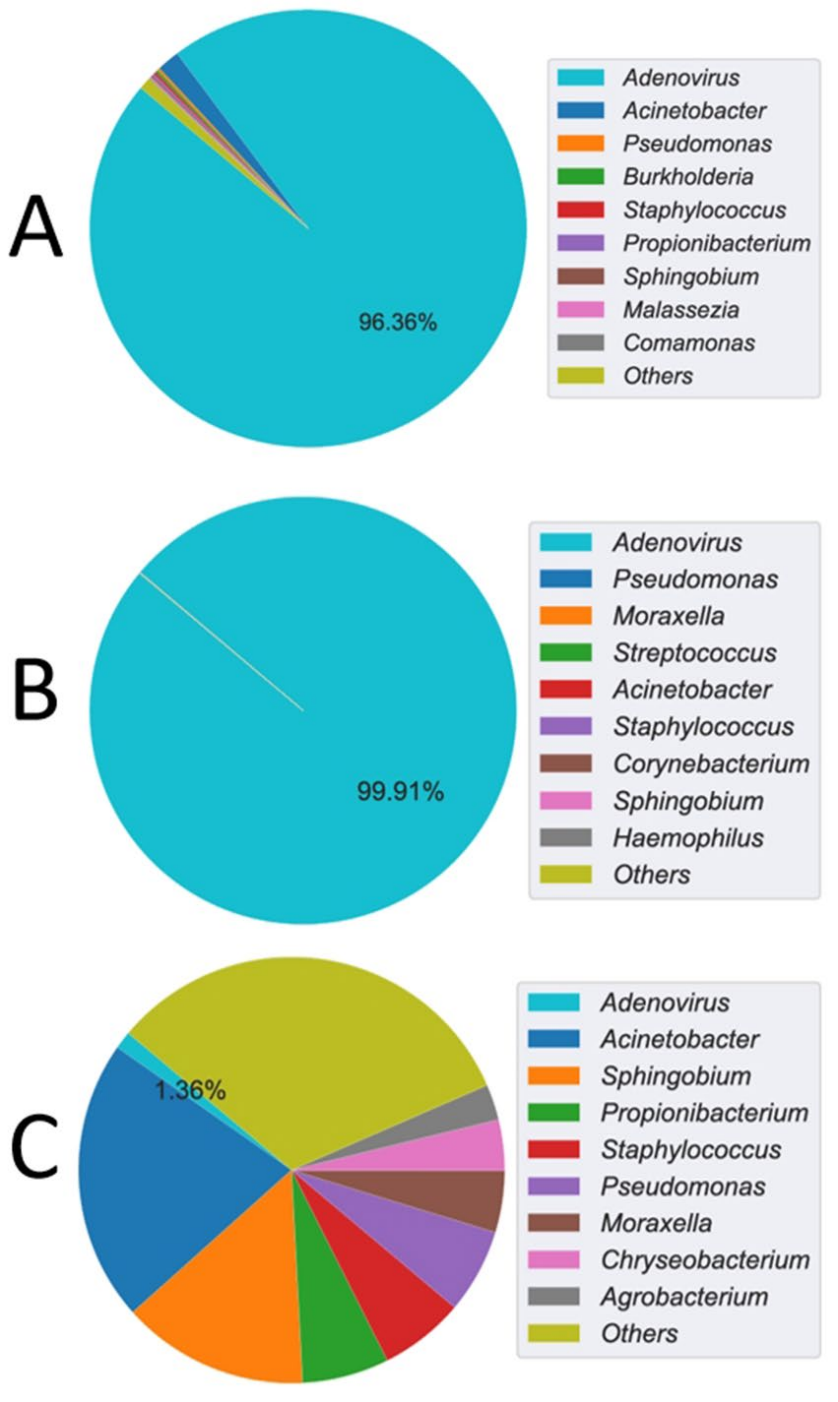
mNGS detection of adenovirus read counts and relative abundance. **(A)** The cerebrospinal fluid (CSF) sample showed an Adenovirus reads per million (RPM) value of 889.7, with a relative abundance of 96.36% among identifiable microbial reads. **(B)** After probe capture enrichment sequencing, the CSF sample revealed a significantly elevated RPM value of 159,492.5 for Adenovirus, corresponding to a relative abundance of 99.91%. **(C)** In contrast, blood mNGS yielded a lower RPM value of 0.8 for Adenovirus, with a relative abundance of 1.36%.

The mNGS analysis of the CSF sample detected a high number of human adenovirus reads, with an reads per million (RPM) value of 889.7 and a relative abundance of 96.36% among identifiable microbial reads (Figure 2A). To obtain deeper coverage, probe-based capture sequencing was subsequently performed on the CSF sample, which resulted in a substantial increase in adenovirus reads, with an RPM value of 159,492.5 and a relative abundance of 99.91% (Figure 2B). Despite the high abundance of adenovirus reads, they could not be accurately matched to any known adenovirus types, suggesting the presence of a novel human adenovirus type. In contrast, the mNGS analysis of the blood sample revealed a lower RPM value of 0.8 for adenovirus, with a relative abundance of 1.36% (Figure 2C). No other viral reads were detected in either the CSF or blood mNGS results, with the remaining reads primarily representing common reagent contaminants, and no other significant pathogens were identified.

### Genomic and phylogenetic analysis

**Figure 3:**
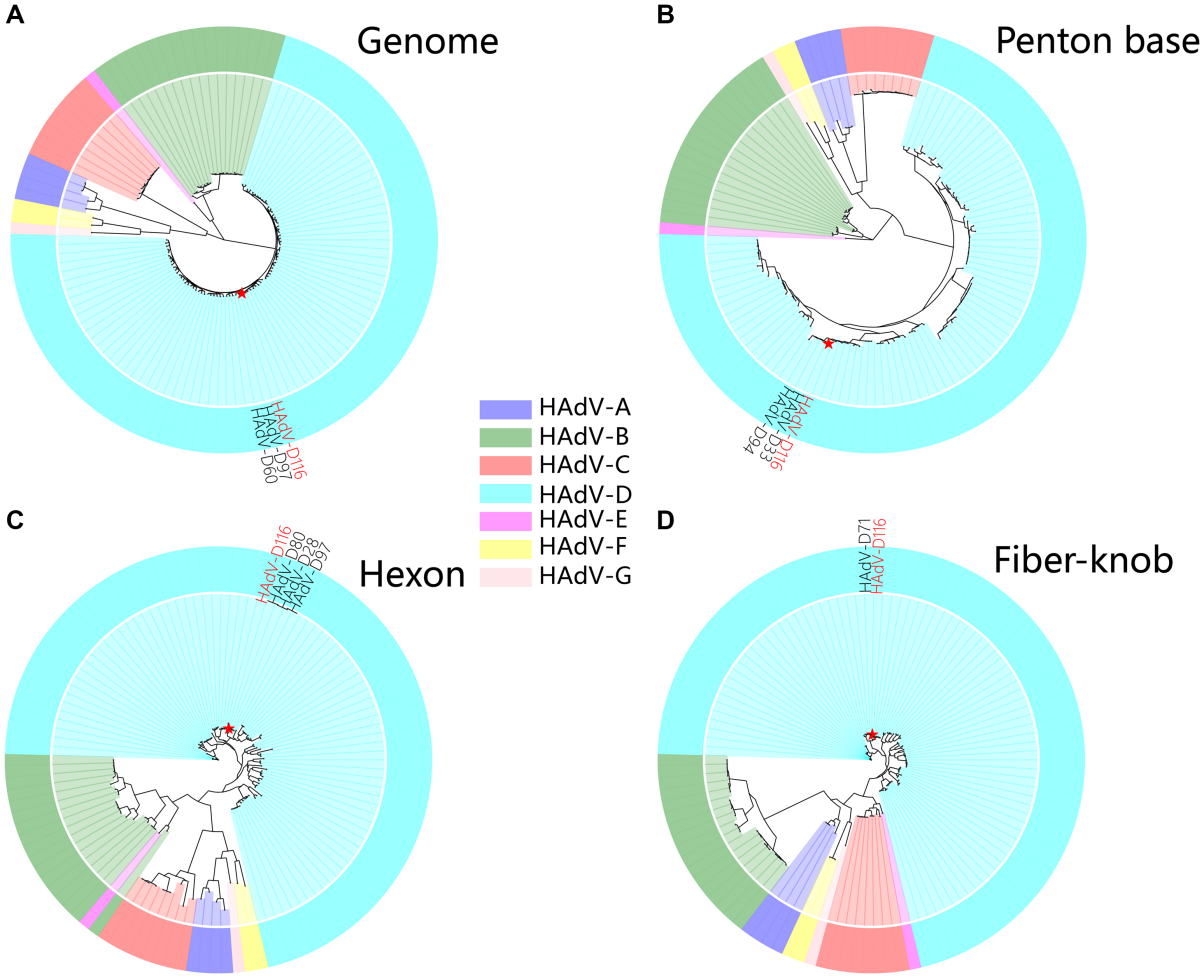
Phylogenetic analysis of HAdV-D116. **(A)** The genome phylogenetic tree was constructed using HAdV-D116 and 112 known HAdV genomes. Multiple alignments were built using Clustal-Omega (v1.2.4). The phylogenetic trees were subsequently constructed with RAxML-NG (v1.2.0), applying the maximum likelihood method with 1,000 bootstrap replicates and the GTR + I + G4 model identified by ModelTest-NG (v0.1.7). Amino acid sequences of penton base **(B)**, hexon **(C)**, and fiber-knob **(D)** were extracted from the genomes, aligned using MUSCLE (v5.2), and the phylogenetic tree was constructed with IQ-TREE (v2.2.2.6) using 1,000 ultrafast bootstraps, automatic model detection, and default parameters.

**Figure 4:**
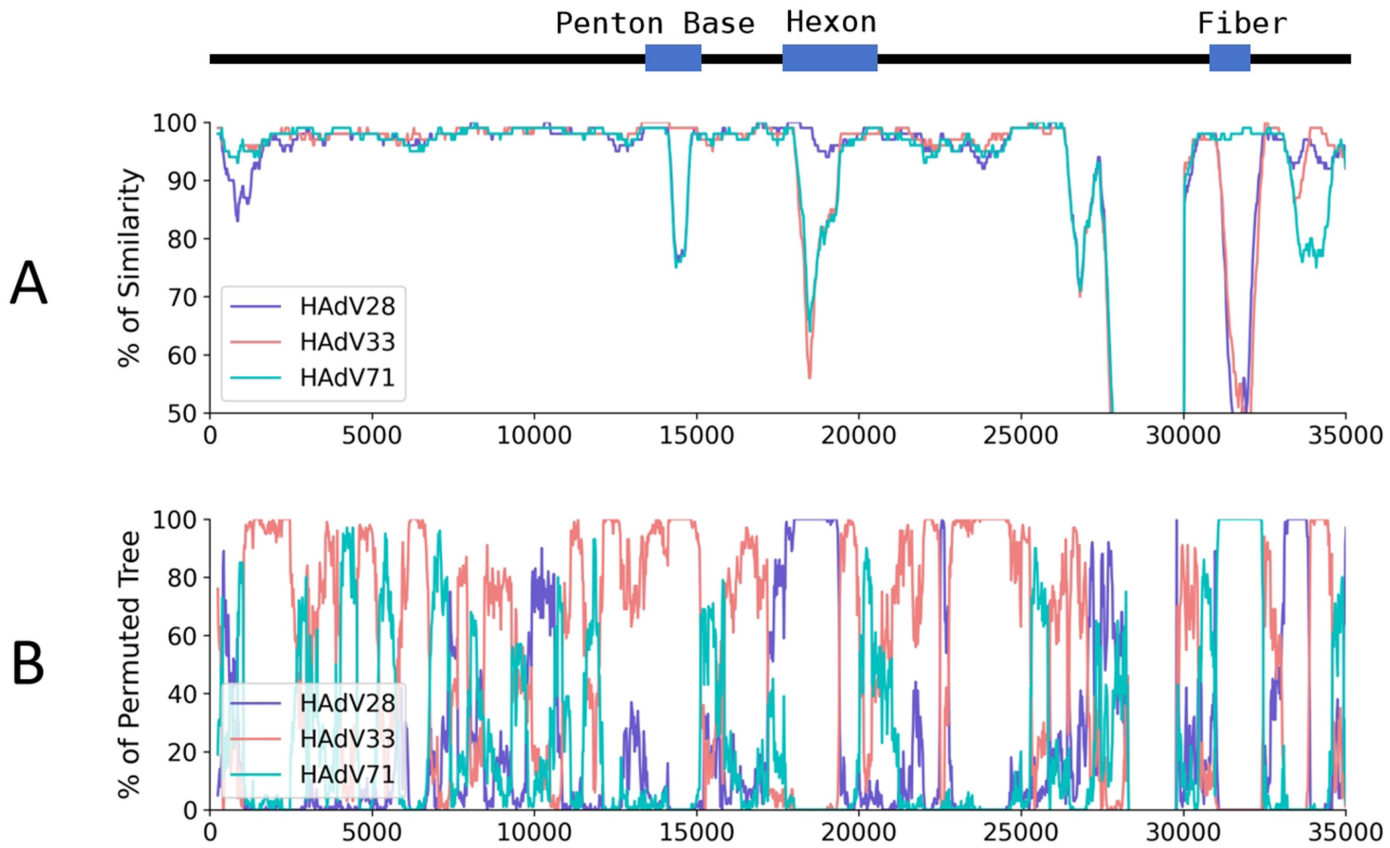
Genome similarity and BootScan analysis of the HAdV-D116 genome. HAdV-D116 was analyzed with HAdV-D33, 28 and 71 using SimPlot (v3.5.1). **(A)** Similarity analysis using HAdV-D116 as query, window is 500 bp and step is 20 bp, other parameters are default. **(B)** BootScan analysis using HAdV-D116 as query, window is 500 bp and step is 20 bp, other parameters are default.

A super-contig of 33,017 base pairs (bp) was assembled from the mNGS data. BLASTn alignment against the nucleotide database identified HAdV-D97, a member of HAdV-D, as the closest match, with a coverage of 97% and an identity of 96.49%. However, several other closely related adenovirus types showed similar levels of coverage and identity, as detailed in Supplementary Table S2, supporting the hypothesis that this represents a novel adenovirus. The GC content of the contig (58%) was consistent with HAdV-D (57%), and phylogenetic analysis placed it within the HAdV-D clade, closely related to HAdV-D97 and HAdV-D60 (Supplementary Figure S1A and Figure 3A). Bootstrap values and phylogenetic distances are depicted in Supplementary Figure S1. Alignment of the penton base (P), hexon (H), and fiber-knob (F) protein sequences of HAdV-D116 to the nucleotide database using BLASTp revealed the highest sequence identities of 99.42, 99.69, and 99.46% with HAdV-D33 (P33), HAdV-D28 (H28), and HAdV-D71 (F71), respectively. These findings were further corroborated by protein phylogenetic analysis (Figures 3B–D and Supplementary Figures S1B–E). SimPlot and BootScan analyses confirmed that this adenovirus genome represents a recombinant of HAdV-D33, HAdV-D28, and HAdV-D71 (Figures 4A,B), leading to the designation of a novel genotype, P33H28F71. This novel adenovirus type has been confirmed and designated HAdV-D116 by the Human Adenovirus Working Group.^6^

### Deletion analysis of HAdV-D116

Although classified within the HAdV-D species, the complete genome of HAdV-D116 (33,017 bp) is significantly shorter than the typical HAdV-D genomes, which average approximately 35,000 bp. Sequence alignment analysis revealed a deletion of approximately 2,000 bp in the E3 gene of HAdV-D116 (beginning at genome position 27,843) compared to HAdV-D60, the closest relative to HAdV-D116 in the phylogenetic tree (Figure 3A). Alignments with other HAdV-D genomes also indicated the presence of large segment deletions in HAdV-D116, with slight variations in deletion lengths depending on the reference genome used.

**Figure 5:**
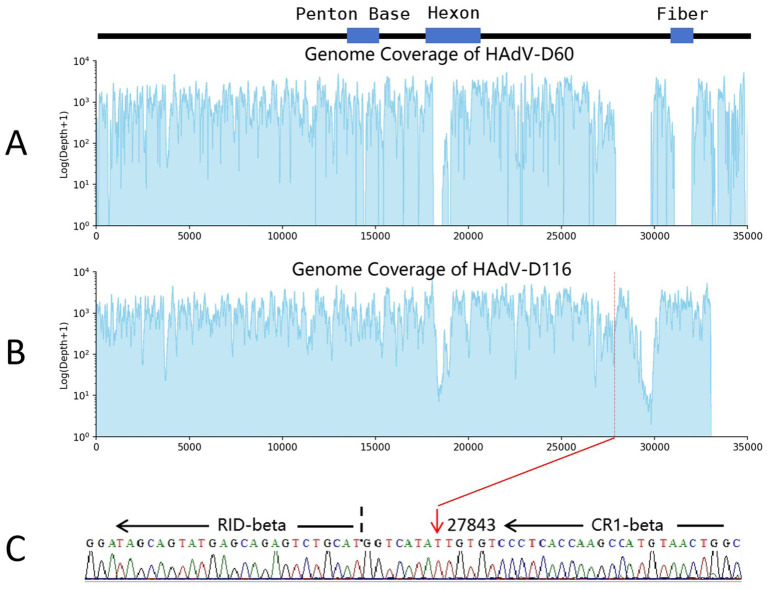
Deletion analysis of HAdV-D116. The cerebrospinal fluid mNGS and hcmNGS datasets were amalgamated and aligned to the genomes of HAdV-D60 and HAdV-D116. **(A)** HAdV-D60 exhibits a lack of read coverage in the region spanning positions 27,914 to 29,808. **(B)** At the presumed starting point (27843) of the deleted fragment, HAdV-D116 demonstrates a depth of 352×. **(C)** Sanger sequencing validation of the large deletion, utilizing the following primer sequences: forward: 5′-GCCAGTTACATGGCTTGGTG-3′ and reverse: 5′-GCAGGAGCAGACCATGACTA-3′, targeting a sequence length of 205 bp (genome positions 27,814–28,018). The red arrow denotes the starting point of the deletion, and the black dotted line represents the RID-beta start site.

To confirm the authenticity of this deletion and rule out the possibility of incomplete assembly, non-human reads from three independent sequencing runs were combined and realigned to both the HAdV-D116 and HAdV-D60 genomes, using HAdV-D60 for comparative analysis. When examining HAdV-D60, no reads mapped to the region corresponding to the putative deletion (Figure 5A). In contrast, the HAdV-D116 genome showed complete read coverage at the deleted loci when visualized using the Integrative Genomics Viewer (IGV), with an average coverage depth of 1,256× across the entire genome and 352× specifically at the putative deletion site (Figure 5B). These findings support the hypothesis of a genuine deletion in HAdV-D116 rather than an assembly artifact. Additional regions in HAdV-D60 also showed an absence of read coverage, likely due to low sequence similarity. Similar results were observed when HAdV-D116 was realigned to other HAdV-D genomes (data not shown).

To further validate the deletion and eliminate the possibility of incomplete sequencing (despite the availability of triplicate datasets), PCR and Sanger sequencing were performed. PCR primers were designed to flank the deletion region, with a 205 bp product expected if the deletion were present, and an approximately 2,000 bp product if the deletion were absent. PCR results confirmed the presence of the 205 bp fragment (Supplementary Figure S3), and Sanger sequencing of this fragment (OR958035) aligned precisely with the expected sequence (Figure 5C). These results conclusively confirm that HAdV-D116 contains a large genomic deletion.

### Genome characteristics of HAdV-D116

**Figure 6:**
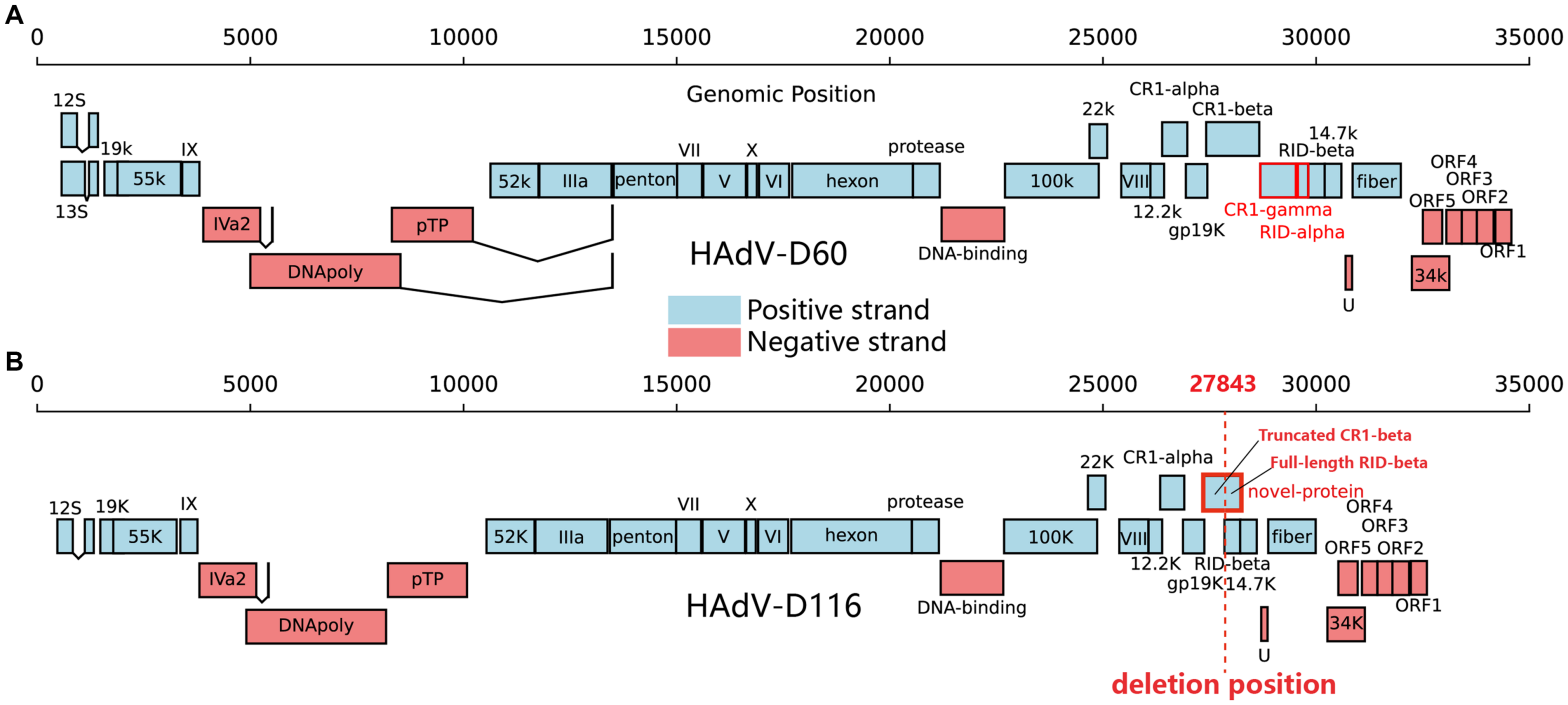
Gene organization in HAdV-D60 and HAdV-D116. The black line above represents the genomic positions. Protein-encoding regions are depicted as boxes. Light blue boxes indicate open reading frames (ORFs) on the positive strand, while light red boxes represent ORFs on the negative strand. **(A)** CR1-gamma and RID-alpha, highlighted in red in HAdV-D60, are the two proteins missing in HAdV-D116. **(B)** The red-framed in HAdV-D116 indicates a novel ORF formed due to the deletion. The red dashed line signifies the starting point of the deletion.

ORF prediction of the HAdV-D116 genome was conducted using ORFfinder, and the resulting amino acid sequences were annotated via BLASTp searches against the nucleotide database. Additionally, VIGOR was employed for further ORF prediction and annotation, with the results verified by BLASTp against the nucleotide database. Upon integrating these findings, a total of 34 ORFs were identified in the HAdV-D116 genome (Supplementary Table S3). Compared to other HAdV-D genomes, the large deletion in the E3 gene of HAdV-D116 resulted in the loss of complete CR1-gamma and RID-alpha, as well as the truncation of CR1-beta. The truncated CR1-beta fused with the complete RID-beta, forming a novel ORF of 870 nucleotides (WQY95711) (Figures 6A,B). BLASTx searches of this novel ORF against the nucleotide database revealed no homologous sequences. However, as experimental validation of the protein product has not been achieved, its existence remains hypothetical.

### Structure analysis of hexon, fiber-knob and penton base proteins

To investigate the potential mechanisms by which HAdV-D116 may infect the CNS, we analyzed the structures of the hexon and fiber-knob proteins, focusing on receptor-binding interactions. Previous studies have shown that HAdV-D56 initiates infection via a direct interaction between the hexon protein and CD46, while HAdV-D26 utilize sialic acid located at the apex of the fiber knob as their adenovirus receptor. Following initial attachment, the penton base protein, through its RGD (Arg-Gly-Asp) motif, interacts with α_v_β_3_ or α_v_β_5_ integrins to facilitate viral internalization via clathrin-mediated endocytosis (Madisch et al., 2007). Protein structure prediction was performed using AlphaFold2, followed by structural alignment through the RCSB online service. Both the hexon and fiber-knob proteins exist as trimers composed of three identical polypeptide chains positioned on the viral capsid surface, and surface electrostatic potential analyses of these trimers were conducted using PyMOL.

**Figure 7:**
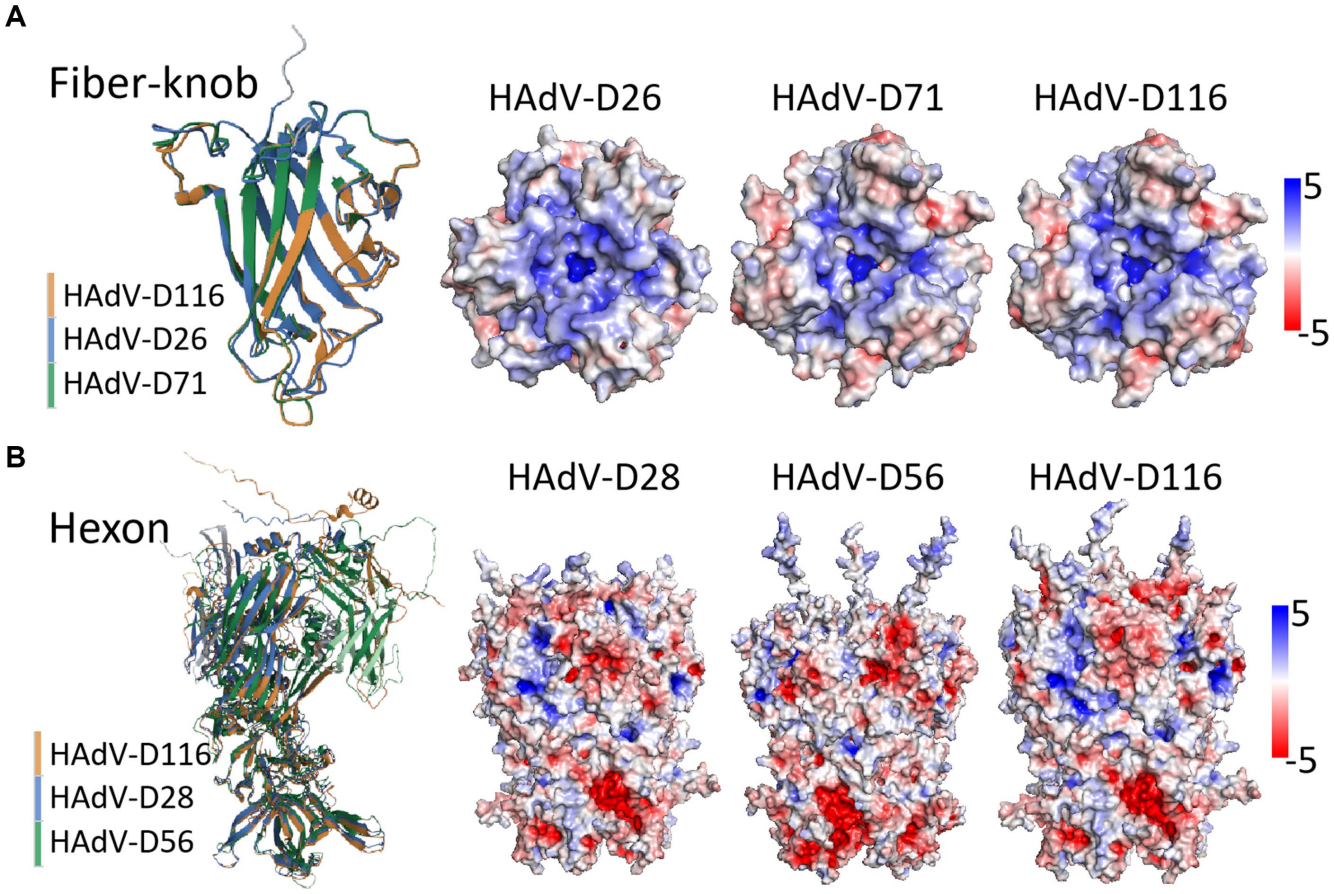
Structure prediction and alignment of hexon and fiber-knob protein. Protein structure prediction was performed using AlphaFold2, with structural alignment via the RCSB online service, and visualization conducted in PyMOL (v2.5.5). **(A)** The fiber-knob structure of HAdV-D116 was compared with those of HAdV-D71 and HAdV-D26, yielding TM-scores of 1 and 0.94, respectively. The trimeric structure reveals the electrostatic potential surfaces in the local basic region within the apical depression—where sialic acid binding occurs—across HAdV-D26, HAdV-D71, and HAdV-D116. **(B)** The hexon structure of HAdV-D116 was compared with those of HAdV-D28 and HAdV-D56, with TM-scores of 0.81 and 0.68, respectively. The trimeric structure and electrostatic potential surfaces are shown for HAdV-D28, HAdV-D56, and HAdV-D116. Electrostatic potential surfaces are displayed at ±5 mV.

The structural prediction for HAdV-D116’s fiber-knob showed high confidence, with a μ-pLDDT of 95.0 ± 6.9 [mean per-residue confidence estimate, where pLDDT values between 70 and 90 indicate “a generally good backbone prediction” and higher values are considered better (Deepmind, and Embl-Ebi, 2022)]. In contrast, the prediction accuracy for the hexon protein was lower, with a μ-pLDDT of 69.7 ± 19.5. The penton base structure prediction showed good confidence with a μ-pLDDT of 81.6 ± 16.1. Analysis of the penton base revealed a well-conserved RGD motif located in a surface-exposed loop (Supplementary Figure S4). Structural alignment revealed that the fiber-knob of HAdV-D116 had a TM-score of 1.0 when compared to HAdV-D71, and 0.94 when compared to HAdV-D26. However, the hexon protein showed lower similarity, with TM-scores of 0.81 and 0.68 relative to HAdV-D28 and HAdV-D56, respectively (Figures 7A,B).

Electrostatic potential analysis of the fiber-knob trimers revealed nearly identical charge distributions between HAdV-D116 and HAdV-D71, with notable similarities to HAdV-D26. Both exhibited a highly basic surface, particularly around the central depression of the fiber-knob apex (Figure 7A). In contrast, the hexon trimer structures and surface charge distributions between HAdV-D116, HAdV-D28, and HAdV-D56 showed significant differences (Figure 7B), possibly due to inaccuracies in structural predictions.

## Discussion

Adenovirus infections of the central nervous system are exceptionally rare, and the specific receptors and mechanisms by which adenoviruses enter neural cells remain poorly characterized. Using mNGS of CSF from a patient with encephalitis and XLA, we identified a novel human adenovirus. Whole-genome phylogenetic analysis classified this adenovirus within the HAdV-D species. Further phylogenetic and recombination analyses of the penton base, hexon, and fiber proteins indicated that this virus is a recombinant, derived from HAdV-D28, HAdV-D33, and HAdV-D71, with the genotype designated as P33H28F71. Of note, it harbors a large ~2000 bp deletion in the E3 region. This novel adenovirus was subsequently confirmed and designated as HAdV-D116 by the Human Adenovirus Working Group.

High read counts for HAdV-D116 were detected in the patient’s CSF, with additional but substantially lower levels observed in the blood. These findings were further validated by qPCR, confirming that HAdV-D116 originated in the CSF, effectively ruling out contamination from blood (Supplementary Table S5). No other viruses or potentially pathogenic microorganisms were detected by mNGS, and traditional microbiological and serological screenings yielded negative results. Based on these findings, viral encephalitis was suspected. Unfortunately, the patient declined further treatment and died 3 weeks later. We attempted to isolate the virus from samples collected during the patient’s lifetime, however, the viral culture was unsuccessful, likely due to suboptimal sample preservation or extended storage duration. Further sampling was not feasible, thus limiting the potential for more in-depth studies that require an isolated viral strain.

To determine whether HAdV-D116 arose from a mixed infection involving HAdV-D28, HAdV-D33, and HAdV-D71 or represents a single viral strain, we realigned non-human reads to the HAdV-D116 genome and conducted variant analysis. The absence of heterozygous variants—expected in the case of a mixed infection due to genomic differences among HAdV-D28, HAdV-D33, and HAdV-D71—ruled out a mixed infection, confirming HAdV-D116 as a single recombinant strain (data not shown). This finding suggests that the recombination event may have occurred prior to infection, rather than within the patient.

Recombination, which can lead to the emergence of novel viruses, often influences tissue tropism and pathogenicity (Walsh et al., 2009). Adenoviruses utilize a wide range of cellular receptors, which are closely tied to their tropism. Unfortunately, all three parental strains of HAdV-D116 (HAdV-D28, HAdV-D33, and HAdV-D71) lack well-documented clinical cases or tissue tropism studies, which limits our understanding of their pathogenic potential (Ismail et al., 2018; Kahl et al., 2010; Robinson et al. 2011b). Generally, adenovirus species enter host cells through interactions between the fiber-knob protein and specific cellular receptors (Stasiak and Stehle, 2020). For example, HAdV-D26 engages sialic acid-bearing glycans via the fiber-knob for cell entry (Baker et al., 2019). Recent studies also indicate that several HAdV-D types, including HAdV-D26, HAdV-D28, and HAdV-D56 can enter cells through a direct interaction between the hexon capsid protein and CD46 (Persson et al., 2021).

Structural predictions and alignments show that HAdV-D116 shares a high TM-score of 0.94 with HAdV-D26 in the fiber-knob region, suggesting substantial similarity (Figure 7A). Additionally, the electrostatic potential of the fiber-knob trimer is nearly identical between HAdV-D116 and HAdV-D26. This structural resemblance suggests that HAdV-D116 may utilize a similar cell entry mechanism, potentially engaging sialic acid-bearing glycans as receptors. Sialic acid-bearing glycans are highly abundant on various cell types, with the brain possessing some of the highest sialic acid content in mammals (Schnaar et al., 2014), which may account for HAdV-D116’s ability to infect the CNS.

CD46, which is also broadly expressed in human tissues, including the brain (Persson et al., 2010), may represent an additional receptor for HAdV-D116. Due to the lower confidence in the hexon protein structure prediction (Figure 7B), it remains uncertain if HAdV-D116 shares structural or functional properties with HAdV-D56’s hexon. However, the hexon gene of HAdV-D116, derived from HAdV-D28, has previously been shown to mediate cell entry through CD46 binding (Persson et al., 2021). Given that CD46 serves as a receptor for numerous HAdV-D types (Persson et al., 2021), it is plausible that HAdV-D116 may employ both fiber-knob binding to sialic acid and hexon interactions with CD46 for cell entry. This dual receptor potential could explain its altered tropism, possibly facilitating infection of CNS tissues.

The penton base protein plays a crucial role in adenovirus internalization through its interaction with cellular integrins. Following the initial attachment mediated by fiber or hexon proteins, the RGD motif in the penton base engages with αv integrins, triggering viral internalization via clathrin-mediated endocytosis (Madisch et al., 2007). Our structural analysis of HAdV-D116’s penton base revealed conservation of the RGD motif, implying that HAdV-D116 likely shares similar entry mechanisms with other HAdV-D types. However, the role of penton base in determining HAdV-D tissue tropism remains unclear, as studies examining this relationship within the HAdV-D species are limited. Further investigation is needed to understand whether penton base variations contribute to the distinct tissue specificity observed among HAdV-D.

**Figure 8:**
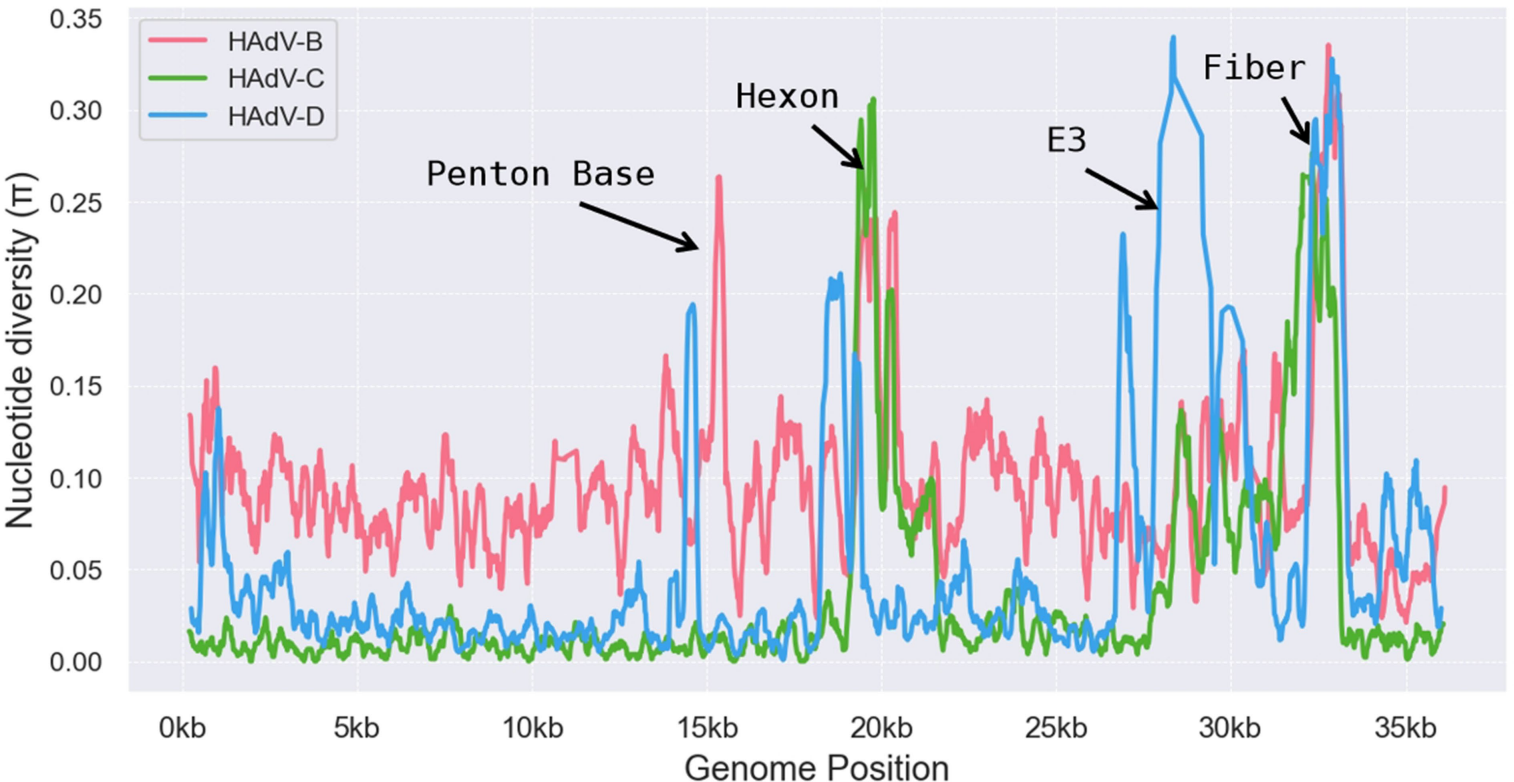
Genomic polymorphism analysis on HAdV-B, HAdV-C, and HAdV-D. Nucleotide diversity plots generated with DnaSP v6 show the average nucleotide differences per site across each HAdV species type. The *y*-axis represents percent diversity, while the *x*-axis denotes nucleotide positions along the genome.

The HAdV-D genome exhibits substantial genetic diversity and is the most populous adenovirus species, with many new types emerging through recombination (Robinson et al., 2013). We conducted a genomic polymorphism analysis of the HAdV-B, C, and D species, which revealed significantly higher polymorphism levels in specific regions, including the penton base, hexon, E3, and fiber genes (Figure 8). Among these, HAdV-B, C, and D subtypes demonstrated pronounced polymorphisms in the hexon and fiber regions, while HAdV-B and HAdV-D displayed considerable genetic variability in the penton base region. Notably, HAdV-D showed the highest degree of polymorphism in the E3 region, with the large deletion (~2000 bp) observed in the E3 gene of HAdV-D116 likely related to this diversity. The variability in the E3 region suggests that these proteins may play a role in defining tissue tropism unique to specific adenovirus types (Burgert and Blusch, 2000), potentially explaining HAdV-D116’s CNS tropism.

The E3 coding region of HAdV-D contains up to eight potential open reading frames (ORFs). Known E3 gene products aid immune evasion but are not required for viral replication *in vitro* (Robinson et al., 2013), a finding consistent with our results. The large deletion in HAdV-D116’s E3 gene did not impair its replication, as evidenced by high read counts in cerebrospinal fluid (CSF) detected by mNGS, indicating extensive replication in the patient’s CSF. Large deletions in the E3 gene have been reported in HAdV-B (Marinheiro et al., 2011; Su et al., 2011), and our study is the first to document such a deletion in HAdV-D. The approximately 2000 bp deletion in the E3 gene led to the complete loss of CR1-gamma and RID-alpha and truncation of CR1-beta, which fused with the complete RID-beta to form a novel ORF of 870 nt. Despite the deletion, the complete RID-beta ORF remained, potentially allowing for normal protein expression. CR1-gamma may play a role in the later stages of the viral replication cycle and in modifying host proteins (Robinson et al., 2011c). RID-alpha is a multifunctional protein that promotes viral infection and replication by regulating cell surface receptor expression, inhibiting apoptosis, and modulating immune responses. These functions make RID-alpha a key factor in helping adenoviruses evade the host immune system and establish persistent infections (Windheim et al., 2004; Horwitz, 2004; Krajcsi et al., 1996; Fessler et al., 2004; Lichtenstein et al., 2004). The CR1-beta (E3/49K) protein, unique to HAdV-D, is a highly glycosylated type I transmembrane protein that binds CD45, leading to CD45 dimerization, which inhibits T-cell and natural killer cell activation, thereby mediating immune regulation within the host (Windheim et al., 2004; Windheim et al., 2013).

These findings suggest that while the absence of CR1-gamma, RID-alpha, and CR1-beta did not impair HAdV-D116’s replication capacity, it may have substantially modified the virus’s interactions with the host immune system and influenced certain biological traits, such as tissue tropism and virulence. There was a possibility that the patient’s fatal encephalitis had been a result of HAdV-D116’s potential CNS tropism and enhanced virulence.

The novel ORF identified in HAdV-D116 lacks homologs in the nucleotide database, and it remains unconfirmed whether this ORF encodes a functional protein. The predicted protein is 290 amino acids in length, consisting of the first 163 amino acids from CR1-beta, two additional residues (164 N and 165 M) derived from six nucleotides upstream of RID-beta’s start codon, followed by the full-length RID-beta (Figure 5C).

Structural predictions indicate local similarities between this novel protein and the CR1-beta and RID-beta proteins of HAdV-D60, suggesting that the fusion did not disrupt original protein domains or create new ones, possibly allowing it to retain partial functions of both CR1-beta and RID-beta (Supplementary Figures S2A–D). The complete CR1-beta protein of HAdV-D60, however, is 414 amino acids long and functions through proteolytic processing. It is initially synthesized as an 80–100 kDa type I transmembrane protein and then cleaved into a large N-terminal fragment (~90 kDa) and a C-terminal fragment (10–13 kDa), with the large ectodomain (sec49K) secreted to bind CD45 (Windheim et al., 2004; Windheim et al., 2013). Even if the novel ORF is expressed as a protein, its truncated CR1-beta segment may be insufficient for processing and secretion, potentially preventing it from forming a ~ 90 kDa N-terminal fragment. The specific function of this novel ORF remains unknown, and experimental validation is needed to confirm this hypothesis.

Adenoviruses are widely used as vectors in vaccine and gene therapy trials due to their broad tissue tropism and ease of manipulation, with HAdV-D, including HAdV-D26, serving as a preferred vector in vaccine development (Baker et al., 2019). Given HAdV-D116’s potential CNS tropism, it could be evaluated as a candidate vector for therapies targeting CNS-related diseases or vaccines aimed at CNS pathogens. Unfortunately, we were unable to isolate a live strain of HAdV-D116. Many HAdV-D types, particularly newly discovered strains, appear to be incidental infections, as they have not been consistently detected in multiple patients, suggesting that they may only affect isolated individuals without further spread. However, if HAdV-D116 is detected again in future surveillance or clinical cases, its unique genomic features would make it an important subject for further research. Such studies would allow us to confirm its potential CNS tropism and examine the role of its E3 gene deletion in greater detail. Moreover, HAdV-D116 could serve as a valuable tool for gene therapy and vaccine development or provide insights for optimizing other adenovirus vectors.

In the post-COVID era, the importance of monitoring emerging pathogens, including adenoviruses, has increased, especially with respect to potential “Disease X” scenarios. Adenoviruses have caused notable outbreaks, such as EKC, underscoring their potential to drive emergent diseases. Our study suggests that recombination events and large deletions in the E3 gene could give rise to novel adenoviruses with altered tissue tropism and potentially increased virulence, posing a particular threat to immunocompromised individuals and potentially leading to wider outbreaks of “Disease X” in the general population. Continuous surveillance and research on novel adenoviruses are therefore essential.

## Data Availability

The mNGS data generated in this study, following the removal of human reads, have been submitted to NCBI under BioProject PRJNA1184469. Accession numbersfor HAdV genomes analyzed in this study are provided in Supplementary Table S4. The accession number for HAdV-D116 is OR958034, and the Sanger sequencesconfirming the large genomic deletion are available under accession numberOR958035.

## Footnotes

^1^ http://hadvwg.gmu.edu/

^2^ https://blast.ncbi.nlm.nih.gov/Blast.cgi

^3^ https://www.ncbi.nlm.nih.gov/orffinder/

^4^ https://colab.research.google.com/github/sokrypton/ColabFold/blob/main/AlphaFold2.ipynb

^5^ https://www.rcsb.org/alignment

^6^ http://hadvwg.gmu.edu/

## Supplementary material

SUPPLEMENTARY FIGURE S1 Phylogenetic analysis of HAdV-D116. **(A)** The genome phylogenetic tree was constructed using HAdV-D116 and 112 known HAdV genomes. Multiple alignments were built using Clustal-Omega (v1.2.4). The phylogenetic trees were subsequently constructed with RAxML-NG (v1.2.0), applying the maximum likelihood method with 1,000 bootstrap replicates and the GTR+I+G4 model identified by ModelTest-NG (v0.1.7). Amino acid sequences of penton base **(B)**, hexon **(C)**, fiber **(D)**, and fiber-knob **(E)** were extracted from the genomes, aligned using MUSCLE (v5.2), and the phylogenetic tree was constructed with IQ-TREE (v2.2.2.6) using 1000 ultrafast bootstraps, automatic model detection, and default parameters.

SUPPLEMENTARY FIGURE S2 Structure prediction of the novel protein. **(A)** The predicted structure of the novel protein in HAdV-D116 retains a β-sheet domain of CR1-beta (indicated by the black arrow), whereas the intact CR1-beta in HAdV-D60 corresponds to two structurally similar β-sheet domains **(B)**. Additionally, the part of novel protein derived from RID-beta also forms an independent domain and is similar to RID-beta of HAdV-D60 **(C)**. **(D)** Structural comparison of novel protein of HAdV-D116 and CR1-beta and RID-beta of HAdV-D60.

SUPPLEMENTARY FIGURE S3 Agarose gel electrophoresis analysis of PCR products validating the HAdV-D116 genomic deletion. PCR amplification was performed using four primer sets (P1-P4) on CSF samples (left panel) and no-template negative controls (NC, right panel), with molecular weight markers (M, 100–2000 bp) indicated on both ends. The primers were designed to validate the deletion: P1 primers (Forward: GCCAGTTACATGGCTTGGTG; Reverse: GCAGGAGCAGACCATGACTA) were designed to flank the deletion site, with an expected product of 205 bp if the deletion is present. This amplicon was used for subsequent Sanger sequencing validation. P2 primers (Forward: TCCTCGCTGATGATTACTCTAAAT; Reverse: AACTGATTTCTGCGGGGAGG) feature a forward primer designed at the deletion junction, expected to generate a 262 bp product only if the deletion exists. P3 primers (Forward: CCTGCGCCAAAAACCAGAAA; Reverse: TGCATTTAGAGTAATCATCAGCGAG) utilize a reverse primer designed at the deletion junction, with an expected product of 196 bp specifically amplifying in the presence of the deletion. P4 primers (Forward: ACTGCAAATTTAACTTCGCTTGC; Reverse: AGGAAGAACCTAGGAATCATGGC) target a conserved region of HAdV-D within the predicted deletion region, with an expected product of 290 bp that should only amplify if the deletion is absent. Analysis of CSF samples revealed distinct bands of expected sizes for P1-P3 (205 bp, 262 bp, and 196 bp), confirming successful amplification across the deletion region. The absence of amplification with P4 primers in CSF samples corroborates the deletion of this conserved region. No amplification was observed in any negative control reactions, confirming the absence of contamination. These PCR results, in conjunction with Sanger sequencing data (Figure 5C), provide independent validation of the HAdV-D116 deletion.

SUPPLEMENTARY FIGURE S4 Structure prediction of HAdV-D116 penton base protein. The predicted structure of HAdV-D116 penton base protein is shown, with the RGD motif highlighted in red.
