# Supplementary figures and images for: Identification and characterization of a novel human adenovirus type HAdV-D116
Source: Front Microbiol. 2025 May 7;16:1566316. doi: 10.3389/fmicb.2025.1566316 (PMC12093491; doi:10.3389/fmicb.2025.1566316)

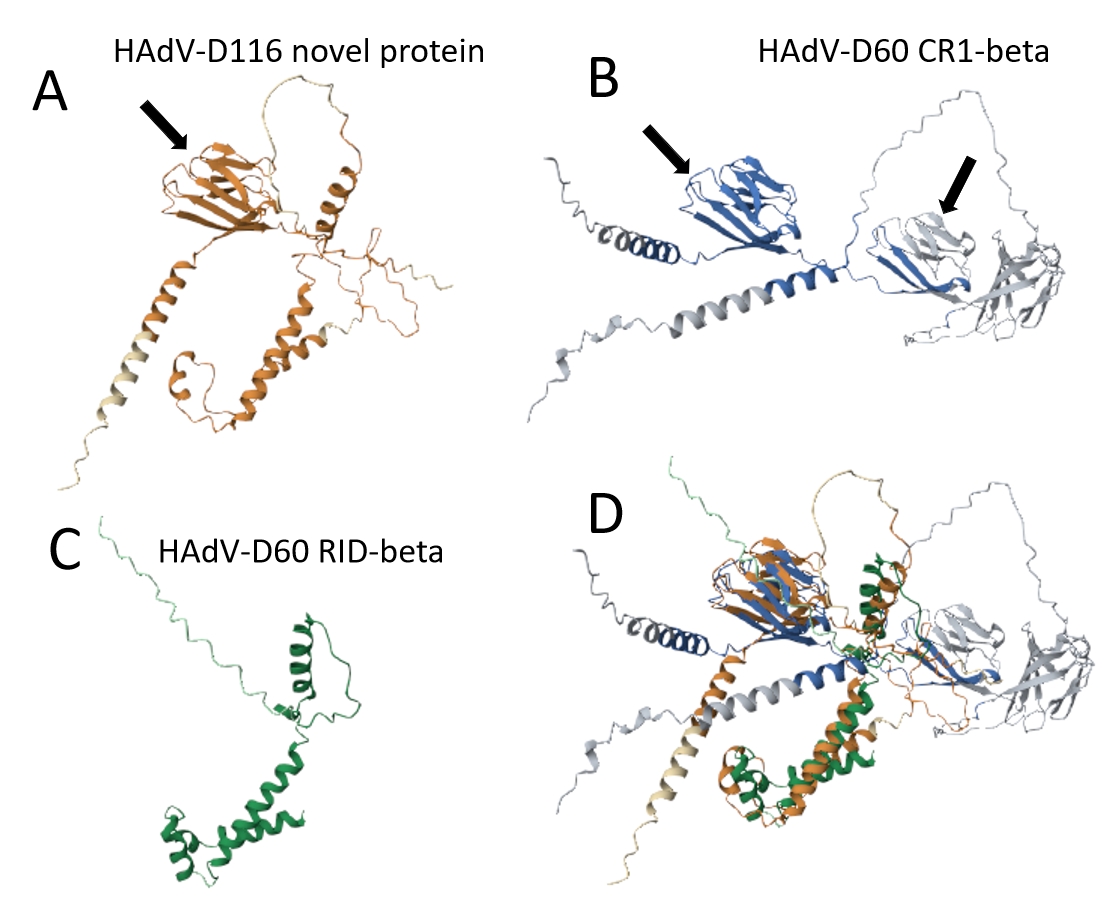

Supplement: SUPPLEMENTARY FIGURE S2 — Structure prediction of the novel protein. (A) The predicted structure of the novel protein in HAdV-D116 retains a β-sheet domain of CR1-beta (indicated by the black arrow), whereas the intact CR1-beta in HAdV-D60 corresponds to two structurally similar β-sheet domains (B). Additionally, the part of novel protein derived from RID-beta also forms an independent domain and is similar to RID-beta of HAdV-D60 (C). (D) Structural comparison of novel protein of HAdV-D116 and CR1-beta and RID-beta of HAdV-D60. [file Image_2.jpeg]

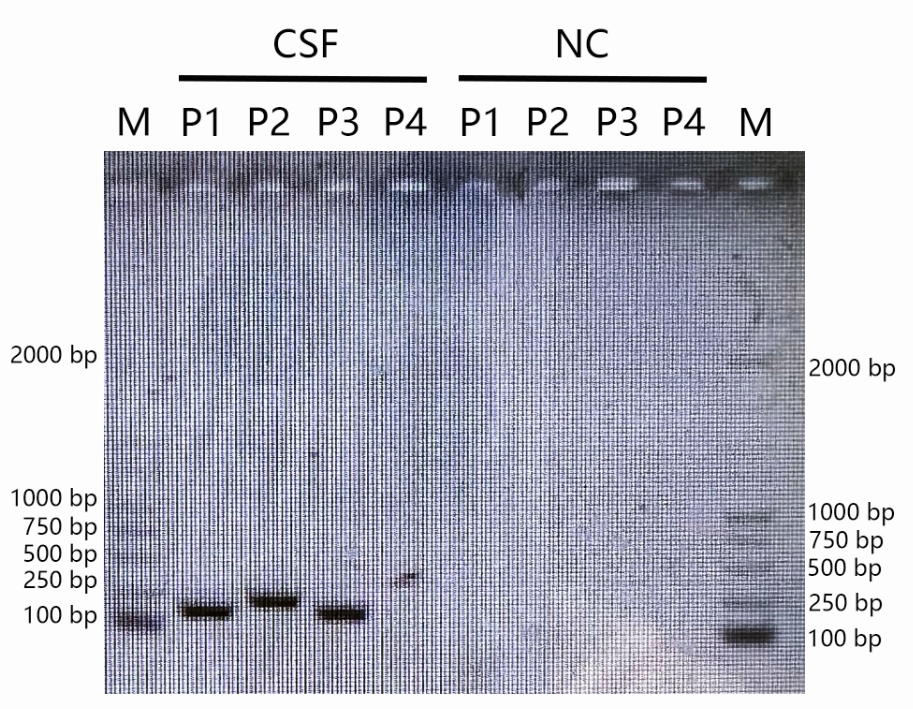

Supplement: SUPPLEMENTARY FIGURE S3 — Agarose gel electrophoresis analysis of PCR products validating the HAdV-D116 genomic deletion. PCR amplification was performed using four primer sets (P1-P4) on CSF samples (left panel) and no-template negative controls (NC, right panel), with molecular weight markers (M, 100–2000 bp) indicated on both ends. The primers were designed to validate the deletion: P1 primers (Forward: GCCAGTTACATGGCTTGGTG; Reverse: GCAGGAGCAGACCATGACTA) were designed to flank the deletion site, with an expected product of 205 bp if the deletion is present. This amplicon was used for subsequent Sanger sequencing validation. P2 primers (Forward: TCCTCGCTGATGATTACTCTAAAT; Reverse: AACTGATTTCTGCGGGGAGG) feature a forward primer designed at the deletion junction, expected to generate a 262 bp product only if the deletion exists. P3 primers (Forward: CCTGCGCCAAAAACCAGAAA; Reverse: TGCATTTAGAGTAATCATCAGCGAG) utilize a reverse primer designed at the deletion junction, with an expected product of 196 bp specifically amplifying in the presence of the deletion. P4 primers (Forward: ACTGCAAATTTAACTTCGCTTGC; Reverse: AGGAAGAACCTAGGAATCATGGC) target a conserved region of HAdV-D within the predicted deletion region, with an expected product of 290 bp that should only amplify if the deletion is absent. Analysis of CSF samples revealed distinct bands of expected sizes for P1-P3 (205 bp, 262 bp, and 196 bp), confirming successful amplification across the deletion region. The absence of amplification with P4 primers in CSF samples corroborates the deletion of this conserved region. No amplification was observed in any negative control reactions, confirming the absence of contamination. These PCR results, in conjunction with Sanger sequencing data (Figure 5C), provide independent validation of the HAdV-D116 deletion. [file Image_3.png]

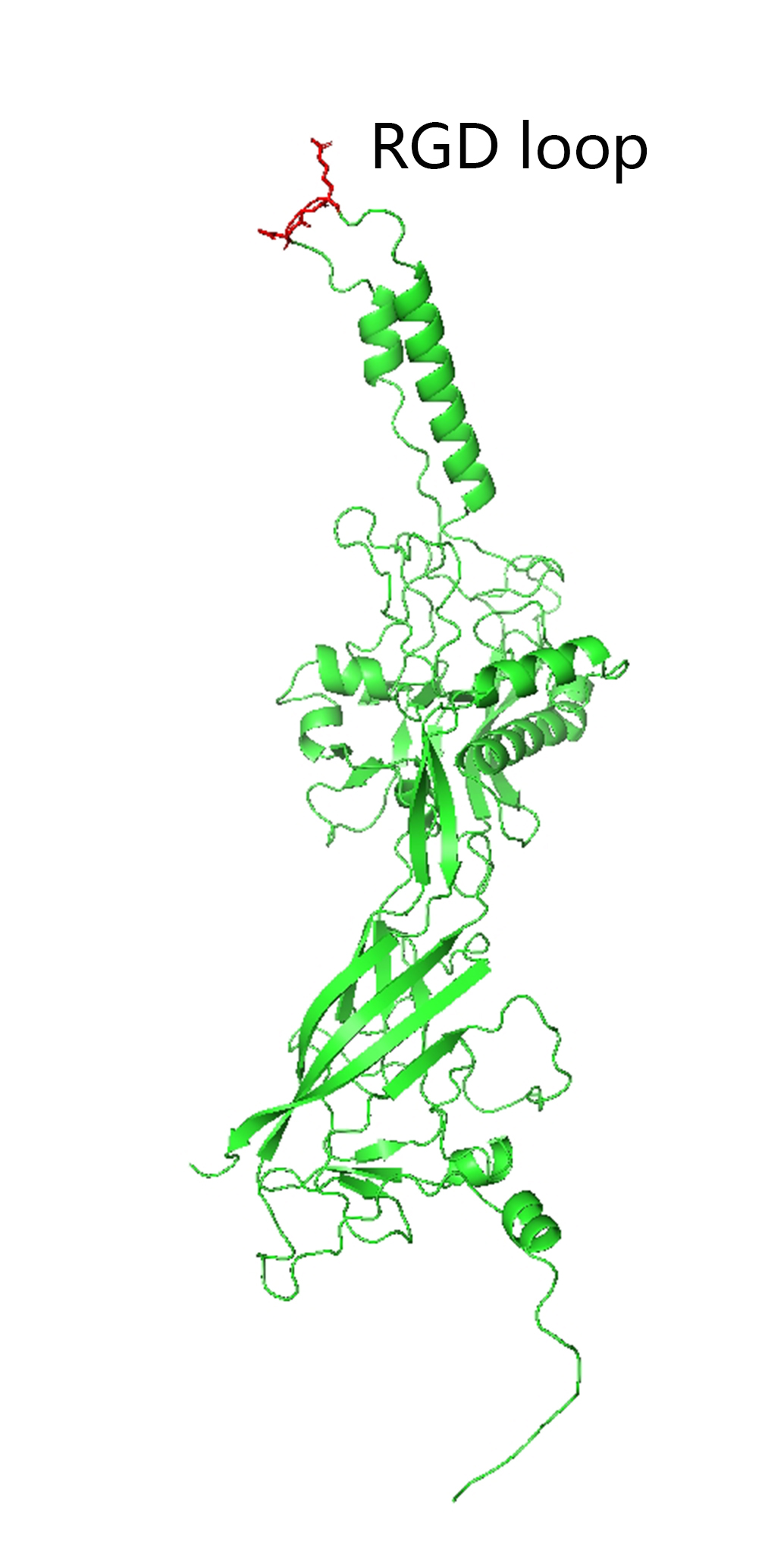

Supplement: SUPPLEMENTARY FIGURE S4 — Structure prediction of HAdV-D116 penton base protein. The predicted structure of HAdV-D116 penton base protein is shown, with the RGD motif highlighted in red. [file Image_4.jpg]
