# Supplementary material for: Identification and characterization of a novel human adenovirus type HAdV-D116
Source: Front Microbiol. 2025 May 7;16:1566316. doi: 10.3389/fmicb.2025.1566316 (PMC12093491; doi:10.3389/fmicb.2025.1566316)
Supplement: Supplementary file 5 [file Table_1.DOCX]

**Supplementary materials**

**Supplementary Table S1. Laboratory data on admission.**

| **Variable** | **On adimission** | **Reference range** |
| --- | --- | --- |
| **Blood** |  |  |
| White cell count (×10^9^/L) | 16.97 | 3.5-9.5 |
| Platelet count (×109/L) | 354 | 100-350 |
| Neutrophils (%) | 78.2 | 50-75 |
| Lymphocytes (%) | 16.3 | 20-40 |
| C-reactive protein (mg/L) | 1.35 | ≤8 |
| Erythrocyte sedimentation rate (mm/hr) | 1 | 0-15 |
| Procalcitonin (ng/m) | <0.072 | - |
| Immunoglobulin G | 7.37 | 7-17 |
| Immunoglobulin A | 0.02 | 0.7-4 |
| Immunoglobulin A | 0.02 | 0.4-2.3 |
| **Cerebrospinal fluid** |  |  |
| Appearrance | Light pink | - |
| Turbidity | Slight | - |
| Total cell count (×10^6^/L) | 1447 | - |
| White cell count (×10^6^/L) | 46 | 0-8 |
| Neutrophils (%) | 8.7 | - |
| Lymphocytes (%) | 91.3 | - |
| Protein (g/L) | 0.94 | 0.15-0.45 |
| Glucose (mmol/L) | 2.5 | 2.5-4.5 |
| lactic acid (mmol/L) | 1.9 | 0.6-2.2 |
| Interleukin-6 (pg/mL) | 277 | <5.9 |
| Interleukin-8 (pg/mL) | 11681 | <62 |
| Interleukin-10 (pg/mL) | 33.2 | <9.1 |

**Supplementary Table S2. The top 10 BLAST result of HAdV-D115 ranked by bit score (Max Score).**

| **Description** | **Scientific Name** | **Max Score** | **Total Score** | **Query Cover** | **E value** | **Per. ident** | **Acc. Len** | **Accession** |
| --- | --- | --- | --- | --- | --- | --- | --- | --- |
| Human mastadenovirus D strain human/DEU/HEIM_00087/1987/97[P67H28F60] | Human mastadenovirus D | 46001 | 52437 | 97% | 0 | 96.49 | 34990 | KF268320.1 |
| Human adenovirus 112 strain D112, complete genome | Human adenovirus 112 | 45683 | 53570 | 98% | 0 | 96.69 | 35112 | OQ679041.1 |
| Human mastadenovirus D isolate human/DEU/Berlin/2014/80[P19/23H28F22] | Human mastadenovirus D | 45624 | 52129 | 96% | 0 | 96.64 | 35145 | KY618679.1 |
| Human adenovirus D strain human/DEU/HEIM_00082/1988/15[P15H15F15], complete genome | Human mastadenovirus D | 45594 | 52232 | 97% | 0 | 96.22 | 35100 | KF268204.1 |
| Human adenovirus 15 DNA, complete genome, isolate: CH38 | Human adenovirus 15 | 45583 | 52193 | 97% | 0 | 96.21 | 35116 | AB562586.1 |
| Human mastadenovirus D strain human/DEU/HEIM_00080/1982/94[P33H15F9] | Human mastadenovirus D | 45417 | 52062 | 97% | 0 | 96.54 | 35082 | KF268201.1 |
| Human mastadenovirus D isolate 592277, complete genome | Human mastadenovirus D | 45148 | 51913 | 98% | 0 | 96.34 | 35084 | OK188910.1 |
| Human adenovirus 67 DNA, complete genome, strain: BGD/2005/B109/[P67/H9/F67] | Human adenovirus 67 | 45144 | 53087 | 98% | 0 | 96.36 | 35075 | AP012302.1 |
| Human adenovirus D109 isolate 592275, complete genome | Human adenovirus D109 | 45142 | 51908 | 98% | 0 | 96.34 | 35097 | OM830314.1 |
| Human adenovirus D strain human/USA/CDPH/2007/59[P64H25F56], complete genome | Human mastadenovirus D | 45040 | 51944 | 98% | 0 | 96.26 | 35072 | JF799911.1 |

**Supplementary Table S3. Genes and proteins of HAdV-D116**

| **No.** | **Gene** | **Product** | **Position** | **Strand** |
| --- | --- | --- | --- | --- |
| 1 | E1A | 12S | join(471..834,1115..1326) | + |
| 2 | E1B | 19K | 1479..2027 | + |
| 3 |  | 55K | 1784..3271 | + |
| 4 | IX | IX | 3356..3760 | + |
| 5 | IVa2 | IVa2 | join(5137..3804,5428..5416) | - |
| 6 | E2B | DNApoly | 8182..4907 | - |
| 7 |  | pTP | 10080..8227 | - |
| 8 | L1 | 52K | 10540..11664 | + |
| 9 |  | IIIa | 11687..13378 | + |
| 10 | L2 | penton | 13432..14985 | + |
| 11 |  | VII | 14989..15573 | + |
| 12 |  | V | 15606..16601 | + |
| 13 |  | X | 16631..16855 | + |
| 14 | L3 | VI | 16911..17615 | + |
| 15 |  | hexon | 17686..20517 | + |
| 16 |  | protease | 20520..21149 | + |
| 17 | E2A | DNA-binding | 22662..21193 | - |
| 18 | L4 | 100K | 22679..24862 | + |
| 19 |  | 22K | 24648..25058 | + |
| 20 |  | VIII | 25384..26067 | + |
| 21 | E3 | 12.2K | 26068..26388 | + |
| 22 |  | CR1-alpha | 26342..26908 | + |
| 23 |  | gp19K | 26875..27378 | + |
| 24 |  | CR1-beta/RID-beta fusion  (hypothetical protein) | 27353..28225 | + |
| 25 |  | RID-beta | 27851..28225 | + |
| 26 |  | 14.7K | 28218..28607 | + |
| 27 | U | U | 28860..28711 | - |
| 28 | L5 | fiber | 28876..29988 | + |
| 29 | E4 | ORF5 | 30976..30521 | - |
| 30 |  | 34K | 31143..30265 | - |
| 31 |  | ORF4 | 31435..31073 | - |
| 32 |  | ORF3 | 31791..31438 | - |
| 33 |  | ORF2 | 32180..31788 | - |
| 34 |  | ORF1 | 32598..32221 | - |

**Supplementary Table S4. Accession numbers for HAdV genomes analyzed in this study。**

| Type | Abbreviated Name | Species | Accession No. |
| --- | --- | --- | --- |
| Human adenovirus 1 | HAdV-C1 | Human mastadenovirus C | AC_000017 |
| Human adenovirus 2 | HAdV-C2 | Human mastadenovirus C | AC_000007 |
| Human adenovirus 3 | HAdV-B3 | Human mastadenovirus B | NC_011203 |
| Human adenovirus 4 | HAdV-E4 | Human mastadenovirus E | NC_003266 |
| Human adenovirus 5 | HAdV-C5 | Human mastadenovirus C | AC_000008 |
| Human adenovirus 6 | HAdV-C6 | Human mastadenovirus C | FJ349096 |
| Human adenovirus 7 | HAdV-B7 | Human mastadenovirus B | AC_000018 |
| Human adenovirus 8 | HAdV-D8 | Human mastadenovirus D | AB746853 |
| Human adenovirus 9 | HAdV-D9 | Human mastadenovirus D | NC_010956 |
| Human adenovirus 10 | HAdV-D10 | Human mastadenovirus D | JN226746 |
| Human adenovirus 11 | HAdV-B11 | Human mastadenovirus B | AF532578 |
| Human adenovirus 12 | HAdV-A12 | Human mastadenovirus A | NC_001460 |
| Human adenovirus 13 | HAdV-D13 | Human mastadenovirus D | JN226747 |
| Human adenovirus 14 | HAdV-B14 | Human mastadenovirus B | JQ824845 |
| Human adenovirus 15 | HAdV-D15 | Human mastadenovirus D | AB562586 |
| Human adenovirus 16 | HAdV-B16 | Human mastadenovirus B | JN860680 |
| Human adenovirus 17 | HAdV-D17 | Human mastadenovirus D | HQ910407 |
| Human adenovirus 18 | HAdV-A18 | Human mastadenovirus A | GU191019 |
| Human adenovirus 19 | HAdV-D19 | Human mastadenovirus D | JQ326209 |
| Human adenovirus 20 | HAdV-D20 | Human mastadenovirus D | JN226749 |
| Human adenovirus 21 | HAdV-B21 | Human mastadenovirus B | KF528688 |
| Human adenovirus 22 | HAdV-D22 | Human mastadenovirus D | FJ619037 |
| Human adenovirus 23 | HAdV-D23 | Human mastadenovirus D | JN226750 |
| Human adenovirus 24 | HAdV-D24 | Human mastadenovirus D | JN226751 |
| Human adenovirus 25 | HAdV-D25 | Human mastadenovirus D | JN226752 |
| Human adenovirus 26 | HAdV-D26 | Human mastadenovirus D | EF153474 |
| Human adenovirus 27 | HAdV-D27 | Human mastadenovirus D | JN226753 |
| Human adenovirus 28 | HAdV-D28 | Human mastadenovirus D | FJ824826 |
| Human adenovirus 29 | HAdV-D29 | Human mastadenovirus D | JN226754 |
| Human adenovirus 30 | HAdV-D30 | Human mastadenovirus D | JN226755 |
| Human adenovirus 31 | HAdV-A31 | Human mastadenovirus A | AM749299 |
| Human adenovirus 32 | HAdV-D32 | Human mastadenovirus D | JN226756 |
| Human adenovirus 33 | HAdV-D33 | Human mastadenovirus D | JN226758 |
| Human adenovirus 34 | HAdV-B34 | Human mastadenovirus B | AY737797 |
| Human adenovirus 35 | HAdV-B35 | Human mastadenovirus B | AC_000019 |
| Human adenovirus 36 | HAdV-D36 | Human mastadenovirus D | GQ384080 |
| Human adenovirus 37 | HAdV-D37 | Human mastadenovirus D | DQ900900 |
| Human adenovirus 38 | HAdV-D38 | Human mastadenovirus D | JN226759 |
| Human adenovirus 39 | HAdV-D39 | Human mastadenovirus D | JN226760 |
| Human adenovirus 40 | HAdV-F40 | Human mastadenovirus F | NC_001454 |
| Human adenovirus 41 | HAdV-F41 | Human mastadenovirus F | MG925782 |
| Human adenovirus 42 | HAdV-D42 | Human mastadenovirus D | JN226761 |
| Human adenovirus 43 | HAdV-D43 | Human mastadenovirus D | JN226762 |
| Human adenovirus 44 | HAdV-D44 | Human mastadenovirus D | JN226763 |
| Human adenovirus 45 | HAdV-D45 | Human mastadenovirus D | JN226764 |
| Human adenovirus 46 | HAdV-D46 | Human mastadenovirus D | AY875648 |
| Human adenovirus 47 | HAdV-D47 | Human mastadenovirus D | JN226757 |
| Human adenovirus 48 | HAdV-D48 | Human mastadenovirus D | EF153473 |
| Human adenovirus 49 | HAdV-D49 | Human mastadenovirus D | DQ393829 |
| Human adenovirus 50 | HAdV-B50 | Human mastadenovirus B | AY737798 |
| Human adenovirus 51 | HAdV-D51 | Human mastadenovirus D | JN226765 |
| Human adenovirus 52 | HAdV-G52 | Human mastadenovirus G | DQ923122 |
| Human adenovirus 53 | HAdV-D53 | Human mastadenovirus D | FJ169625 |
| Human adenovirus 54 | HAdV-D54 | Human mastadenovirus D | AB333801 |
| Human adenovirus 55 | HAdV-B55 | Human mastadenovirus B | FJ643676 |
| Human adenovirus 56 | HAdV-D56 | Human mastadenovirus D | HM770721 |
| Human adenovirus 57 | HAdV-C57 | Human mastadenovirus C | HQ003817 |
| Human adenovirus 58 | HAdV-D58 | Human mastadenovirus D | HQ883276 |
| Human adenovirus 59 | HAdV-D59 | Human mastadenovirus D | JF799911 |
| Human adenovirus 60 | HAdV-D60 | Human mastadenovirus D | HQ007053 |
| Human adenovirus 61 | HAdV-A61 | Human mastadenovirus A | JF964962 |
| Human adenovirus 62 | HAdV-D62 | Human mastadenovirus D | JN162671 |
| Human adenovirus 63 | HAdV-D63 | Human mastadenovirus D | JN935766 |
| Human adenovirus 64 | HAdV-D64 | Human mastadenovirus D | EF121005 |
| Human adenovirus 65 | HAdV-D65 | Human mastadenovirus D | AP012285 |
| Human adenovirus 66 | HAdV-B66 | Human mastadenovirus B | JN860676 |
| Human adenovirus 67 | HAdV-D67 | Human mastadenovirus D | AP012302 |
| Human adenovirus 68 | HAdV-B68 | Human mastadenovirus B | JN860678 |
| Human adenovirus 69 | HAdV-D69 | Human mastadenovirus D | JN226748 |
| Human adenovirus 70 | HAdV-D70 | Human mastadenovirus D | KP641339 |
| Human adenovirus 71 | HAdV-D71 | Human mastadenovirus D | KF268207 |
| Human adenovirus 72 | HAdV-D72 | Human mastadenovirus D | KF268335 |
| Human adenovirus 73 | HAdV-D73 | Human mastadenovirus D | KY618676 |
| Human adenovirus 74 | HAdV-D74 | Human mastadenovirus D | KY618677 |
| Human adenovirus 75 | HAdV-D75 | Human mastadenovirus D | KY618678 |
| Human adenovirus 76 | HAdV-B76 | Human mastadenovirus B | KF633445 |
| Human adenovirus 77 | HAdV-B77 | Human mastadenovirus B | KF268328 |
| Human adenovirus 78 | HAdV-B78 | Human mastadenovirus B | KT970441 |
| Human adenovirus 79 | HAdV-B79 | Human mastadenovirus B | LC177352 |
| Human adenovirus 80 | HAdV-D80 | Human mastadenovirus D | KY618679 |
| Human adenovirus 81 | HAdV-D81 | Human mastadenovirus D | AB765926 |
| Human adenovirus 82 | HAdV-D82 | Human mastadenovirus D | LC066535 |
| Human adenovirus 83 | HAdV-D83 | Human mastadenovirus D | KX827426 |
| Human adenovirus 84 | HAdV-D84 | Human mastadenovirus D | MF416150 |
| Human adenovirus 85 | HAdV-D85 | Human mastadenovirus D | LC314153 |
| Human adenovirus 86 | HAdV-D86 | Human mastadenovirus D | KX868297 |
| Human adenovirus 87 | HAdV-D87 | Human mastadenovirus D | MF476841 |
| Human adenovirus 88 | HAdV-D88 | Human mastadenovirus D | MF476842 |
| Human adenovirus 89 | HAdV-C89 | Human mastadenovirus C | MH121097 |
| Human adenovirus 91 | HAdV-D91 | Human mastadenovirus D | KF268208 |
| Human adenovirus 92 | HAdV-D92 | Human mastadenovirus D | KF268325 |
| Human adenovirus 93 | HAdV-D93 | Human mastadenovirus D | KF268334 |
| Human adenovirus 94 | HAdV-D94 | Human mastadenovirus D | KF268201 |
| Human adenovirus 95 | HAdV-D95 | Human mastadenovirus D | KF268206 |
| Human adenovirus 96 | HAdV-D96 | Human mastadenovirus D | KF268327 |
| Human adenovirus 97 | HAdV-D97 | Human mastadenovirus D | KF268320 |
| Human adenovirus 98 | HAdV-D98 | Human mastadenovirus D | KF268332 |
| Human adenovirus 99 | HAdV-D99 | Human mastadenovirus D | KF268211 |
| Human adenovirus 100 | HAdV-D100 | Human mastadenovirus D | KF268330 |
| Human adenovirus 101 | HAdV-D101 | Human mastadenovirus D | KF268324 |
| Human adenovirus 102 | HAdV-D102 | Human mastadenovirus D | KF268312 |
| Human adenovirus 103 | HAdV-D103 | Human mastadenovirus D | KF268322 |
| Human adenovirus 104 | HAdV-C104 | Human mastadenovirus C | MH558113 |
| Human adenovirus 105 | HAdV-D105 | Human mastadenovirus D | ON393913 |
| Human adenovirus 106 | HAdV-B106 | Human mastadenovirus B | ON393912 |
| Human adenovirus 107 | HAdV-D107 | Human mastadenovirus D | MK174992 |
| Human adenovirus 108 | HAdV-C108 | Human mastadenovirus C | OQ518326 |
| Human adenovirus 109 | HAdV-D109 | Human mastadenovirus D | OM830314 |
| Human adenovirus 110 | HAdV-D110 | Human mastadenovirus D | OM830315 |
| Human adenovirus 111 | HAdV-D111 | Human mastadenovirus D | LC652931 |
| Human adenovirus 112 | HAdV-D112 | Human mastadenovirus D | OQ679041 |
| Human adenovirus 113 | HAdV-D113 | Human mastadenovirus D | MW694832 |

**Supplementary Table S5: HAdV-D116 qPCR quantification results.**

| **Sample** | **CT Value** | **Copies/ml** | **Viral Load (copies/ml)** |
| --- | --- | --- | --- |
| CSF | 34.37 | 200.07 | 4,001.34 |
|  | 34.67 | 160.56 | 3,211.11 |
|  | 34.57 | 172.77 | 3,455.45 |
| Blood | 35 | – | Below detection limit |
|  | 35 | – | Below detection limit |
|  | 35 | – | Below detection limit |

Note: CSF nucleic acid extract was diluted 20-fold due to limited sample availability; results have been adjusted accordingly (original result ×20). HAdV-D116-specific primers used for qPCR were: Forward primer: CGCACTTTCATTCTCGAGCG; Reverse primer: GGACAAAGTCGGAGGGGAAG.
